# Infection of host plants by Cucumber mosaic virus increases the susceptibility of *Myzus persicae* aphids to the parasitoid *Aphidius colemani*

**DOI:** 10.1038/srep10963

**Published:** 2015-06-04

**Authors:** Kerry E. Mauck, Consuelo M. De Moraes, Mark C. Mescher

**Affiliations:** 1 Department of Environmental Systems Science, ETH Zürich, 8092 Zürich, Switzerland

## Abstract

Plant viruses can profoundly alter the phenotypes of their host plants, with potentially far-reaching implications for ecology. Yet few studies have explored the indirect, host-mediated, effects of plant viruses on non-vector insects. We examined how infection of *Cucurbita pepo* plants by *Cucumber mosaic virus* (CMV) impacted the susceptibility of aphids (*Myzus persicae*) to attack by the parasitoid wasp *Aphidius colemani*. In semi-natural foraging assays, we observed higher rates of aphid parasitism on infected plants compared to healthy plants. Subsequent experiments revealed that this difference is not explained by different attack rates on plants differing in infection status, but rather by the fact that parasitoid larvae successfully complete their development more often when aphid hosts feed on infected plants. This suggests that the reduced nutritional quality of infected plants as host for aphids—documented in previous studies—compromises their ability to mount effective defenses against parasitism. Furthermore, our current findings indicate that the aphid diet during parasitoid development (rather than prior to wasp oviposition) is a key factor influencing resistance. These findings complement our previous work showing that CMV-induced changes in host plant chemistry alter patterns of aphid recruitment and dispersal in ways conducive to virus transmission.

Insect-vectored plant viruses are near-ubiquitous components of natural and agricultural ecosystems and often reach high frequencies within plant populations^1^,^2^,^3^. These pathogens can significantly alter the phenotypes of infected hosts, with important and far-reaching implications for ecology, since variation in plant traits that mediate interactions with other organisms can profoundly influence community interaction webs^4^. Yet, relatively few studies to date have addressed plant virus ecology in a community context^5^,^6^,^7^,^8^,^9^. Furthermore, little is known about the ways in which indirect, host-mediated effects of plant viruses may influence the ecology of non-vector insects, including members of higher trophic levels that exploit herbivorous prey.

Previous work has documented virus effects on various aspects of plant physiology, morphology, and biochemistry that are known to play important roles in interactions among host plants, insect vectors, and other non-vector insects. For instance, several plant viruses have been shown to alter plant volatile emissions that serve as key foraging cues for insect herbivores and their natural enemies^10^,^11^,^12^,^13^,^14^,^15^,^16^,^17^. Viruses can also influence plant defense chemistry, including the constitutive and induced expression of phytohormones that mediate defense responses to herbivory and infection^18^,^19^,^20^,^21^, and alter plant nutritional quality by diverting resources towards virus replication and shifting the allocation of nutrients among host tissues^22^,^23^,^24^. Virus-induced changes in host-plant architecture, size, leaf morphology, and color are also common^25^,^26^. Furthermore, the molecular and biochemical mechanisms underlying these diverse effects of viruses on plants have been elucidated by monitoring gene expression, protein changes, and metabolite profiles of host plants infected by wild-type and genetically modified viruses^18^,^24^,^26^,^27^,^28^,^29^.

From an ecological perspective, most previous work has focused on implications of such virus-induced changes in host plants for interactions between plants and insect vectors and consequently for disease transmission (reviewed in^19^). For example, we previously reported that infection of *Cucurbita pepo* by *Cucumber mosaic virus* (CMV), a widespread pathogen that infects numerous hosts, causes significant changes in host plant chemistry that influence the behavior of aphid vectors (*Aphis gossypii* and *Myzus persicae*) with implications for virus transmission^15^,^17^,^24^,^30^. Specifically, CMV infection causes elevated emission of plant volatiles without eliciting major changes in the composition of the volatile blend, resulting in enhanced attraction of aphid vectors to the odors of infected plants^15^. However, infection also reduces plant nutritional quality for aphids and promoted rapid aphid dispersal from infected plants^15^,^17^,^24^,^30^. The overall pattern of plant-aphid interactions resulting from these effects—enhanced aphid attraction to infected plants followed by rapid dispersal in response to gustatory cues—appears conducive to the transmission of CMV, a non-persistently transmitted virus that is rapidly acquired (and transmitted) by aphids during brief probes of plant epidermal cells^6^,^31^,^32^. Other work indicates that CMV similarly reduces the quality of other host plants for a number of common aphid vectors^15^,^29^,^33^ and that these effects occur under natural conditions^15^.

Relatively few previous studies have examined how physiological changes in host plants induced by viruses influence interactions involving non-vector herbivores and higher trophic levels, or the implications of such effects for the transmission and spread of viruses. Natural enemies can have significant effects on vector (e.g., aphid) behaviors relevant to virus transmission (e.g. by increasing movement between plants and probing) and on the size of vector populations^34^,^35^,^36^,^37^. And non-vector herbivores can have significant effects on plant survival (reviewed in^38^), influencing the time period over which a virus-infected plant persists in the landscape as a source of inoculum. In a recent study examining the effects of CMV infection on the interactions of *C. pepo* with the broader insect community, we found that predators and parasitoids were largely indifferent to virus infection and were just as successful at locating aphid prey on CMV-infected plants as on healthy plants under field conditions^30^. However, non-vector herbivores avoided colonizing and ovipositing on CMV-infected plants, which have drastically altered nutrient levels and reduced palatability, allowing these infected plants to escape much of the extensive herbivore damage incurred by healthy plants in our field study^30^. These results have potentially important implications for aphid ecology and virus transmission, as they suggest that aphid vectors on virus-infected plants will not escape predation (or the sub-lethal effects of disturbance by predators), while virus-infected plants may experience reduced herbivore damage compared to healthy plants.

Building on our previous investigation of the effects of CMV on host-plant phenotype and plant interactions with aphid vectors^15^,^17^,^24^, as well as with the broader insect community^30^, the current study explores the impacts of CMV infection on the ecology of an aphid parasitoid, *Aphidius colemani*, and its success in parasitizing aphids (*Myzus persicae*) on infected hosts. Non-persistently transmitted viruses like CMV do not enter the aphid hemocoel, where the parasitoid develops (being restricted to the aphid gut after ingestion), nor do they appear to have significant direct effects on the physiology of aphid vectors; nevertheless, they may still have significant impacts on the aphid host environment for parasitoids via their effects on host-plant chemistry. A recent review of past studies suggests that non-persistently transmitted viruses frequently have negative effects on plant quality and palatability for vectors^19^, as was shown for CMV^15^,^29^,^33^,^39^. Furthermore, previous studies have also reported that factors that reduce plant quality for insects (e.g. nutrition deficiencies, secondary metabolites and induced defenses) can have either negative effects (e.g.^40^,^41^) or positive effects (e.g.^42^,^43^,^44^) on the performance of parasitoids. To explore how CMV-induced changes in plant quality and volatile emissions influence aphid parasitoids we assessed parasitoid foraging preferences and performance via whole-plant and olfactometer-based behavioral assays as well as no-choice parasitoid oviposition experiments in combination with manipulation of the infection status of aphid host food plants.

## Results

### Parasitoid behavior: semi-natural foraging experiments and olfactometer assays

**Figure 1:**
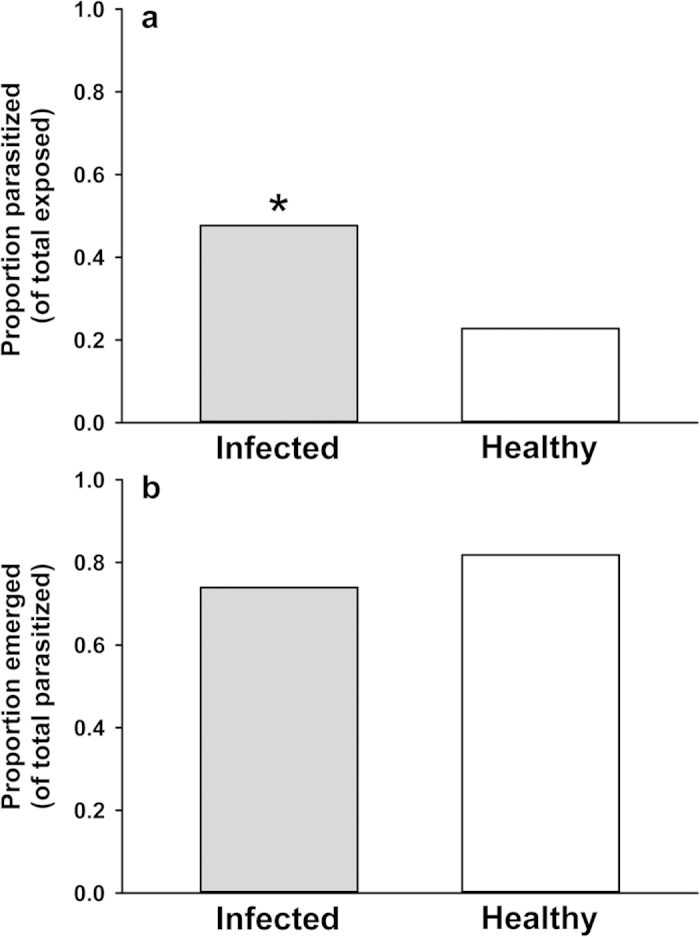
Parasitism and emergence rates from the semi-natural foraging assay. [**a**] Proportion of wasps presented in the foraging assay that developed into mummies (Z = 5.11, two-tailed P = 0.0001). Infected N = 193, healthy N = 193 (aphids exposed to parasitoids). [**b**] Of the mummies that developed, the proportion of mummies for which a wasp successfully completed development (Z = −1.02, two-tailed P = 0.2856). Infected N = 99, healthy N = 44 (mummies tracked to emergence). * indicates significance at P < 0.05. NOTE: bars represent proportions of a total and so cannot have a measure of error.

**Figure 2:**
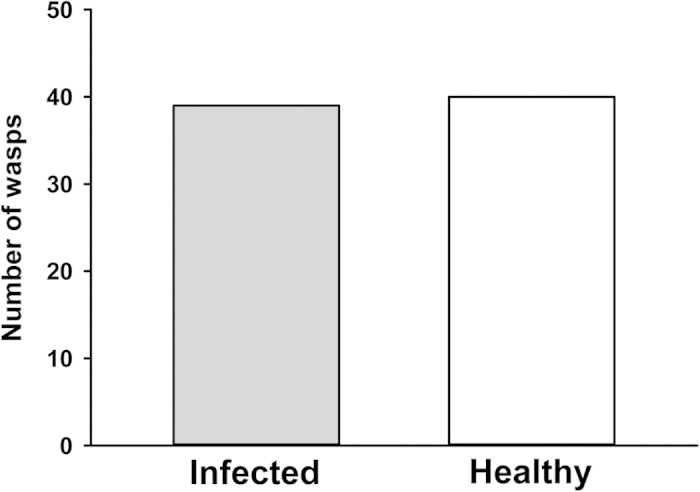
Odor-based foraging by female wasps. Number of wasps choosing each treatment in choice tests with infected and healthy plants housing equal populations of susceptible aphid hosts (df = 1, χ^2^ = 0.013, P = 0.91). Thirteen wasps did not make a choice and were not included in the analysis. NOTE: bars represent proportions of a total and so cannot have a measure of error.

To assess parasitism rates on infected and healthy plants in a semi-natural setting, we employed an assay in which wasps foraged among infected and healthy plants inside in a large mesh cage (1 m^3^). Each plant was colonized with the same number of susceptible, 3^rd^ instar aphid hosts, which originated from small colonies kept on infected or healthy plants for several days prior to the experiment (infected test plants received aphids from infected plant colonies, and healthy test plants from healthy plant colonies). While equal numbers of susceptible aphids were present on plants of each infection status, more pupal mummies subsequently developed on CMV-infected plants than on healthy plants (Fig. 1A). However, no difference in rates of wasp emergence from mummies was observed among infection treatments (Fig. 1B). To explore whether differences in parasitism rates observed in our foraging assay were influenced by differential attraction of female wasps to the odors of virus-infected plants, we used a Y-tube olfactometer to present volatile cues from aphid-infested infected and healthy plants in the absence of contact and visual cues. Wasps showed no preference for the odors of infected plants or healthy plants in these assays, suggesting that the difference in parasitism success rates seen in the semi-natural foraging assay were not due to differential orientation of wasps to CMV-infected plants (Fig. 2).

### Parasitoid performance (developmental success)

**Figure 3:**
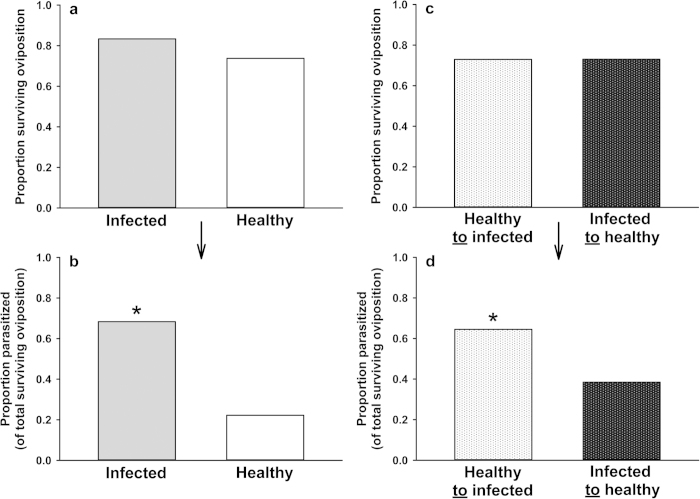
Survival and parasitism rates with host plant treatment held constant or switched following oviposition. [**a**] Proportion of the total number of parasitized aphids surviving three days following oviposition by *A. colemani* where host plant infection status was the same for each aphid before and after oviposition (Z = 1.35, two-tailed P = 0.1802). Infected N = 72, healthy N = 61 (number of aphids exposed to parasitoid oviposition). [**b**] Of those surviving, the proportion that successfully developed into mummies (Z = 4.68, two-tailed P = 0.0001). Infected N = 60, healthy N = 45 (number surviving oviposition). [**c**] Proportion of the total number of parasitized aphids surviving three days following oviposition by *A. colemani* where host plant infection status is switched following oviposition (Z = −0.014, P = 0.99). Infected N = 85, healthy N = 89 (number of aphids receiving parasitoid oviposition). [**d**] Of those surviving, the proportion that successfully developed into mummies (Z = 18.90, P = 0.0001). Infected N = 62, healthy N = 65 (number of aphids surviving oviposition). * indicates significance at P < 0.05. NOTE: bars represent proportions of a total and so cannot have a measure of error.

To determine whether observed differences in parasitism rates on healthy and infected plants were influenced by indirect effects of CMV on aphid quality for the parasitoid we tracked the success of parasitoid development following observed oviposition events. When aphids were reared on either healthy or infected plants, parasitized, and then returned to a plant of the same infection treatment, nearly three times as many mummies developed from aphid hosts living on CMV-infected plants compared to those on healthy plants (Fig. 3A, B). We next conducted a similar experiment but switched aphids to plants of the alternate infection treatment following wasp oviposition (in order to compare the relative influence of aphid feeding on CMV-infected plant tissue prior to parasitism and during parasitoid development). Here we observed that aphid feeding on CMV-infected plants either before or after parasitism yielded some improvement in the rate of parasitoid success relative to continuous aphid feeding on healthy plants; however, this effect was much more pronounced when aphids fed on infected plants during parasitoid development) (Fig. 3C, D).

## Discussion

In assays where *A. colemani* parasitoids were allowed to forage for aphid hosts on healthy and CMV-infected plants under semi-natural conditions, we observed significantly higher rates of parasitism on infected relative to healthy plants, measured as an increase in the number of parasitized aphids progressing to the (pupal) mummy stage (Fig. 1). There was no difference among treatments in the subsequent rate of parasitoid emergence.

Induced plant volatiles are known to play a key role in host location by parasitoids of herbivorous insects, including aphids^45^. However, we observed no significant difference in parasitoid attraction to the volatile emissions of healthy and CMV-infected plants infested with *M. persicae* in Y-tube olfactometer assays (Fig. 2), suggesting that the increased parasitism rates observed in our previous experiments are not explained by enhanced attraction of wasps to the odors of infected plants. We have previously reported that CMV infection causes the elevation of volatile emission rates from *C. pepo*—both in the laboratory and from plants subjected to aphid feeding under field conditions^15^. However, we also noted that these increased emissions are offset by the smaller physical size of infected plants, so that the overall emission profiles of infected plants may resemble those of (larger) healthy plants^15^,^30^. Moreover, while CMV-infected plants exhibit higher rates of volatile emission, the volatile blend that they emit remains qualitatively similar to that of healthy plants^15^,^30^. Thus our previous findings regarding the odor profiles of CMV-infected and healthy plants are consistent with the failure of *A. colemani* females to discriminate between the odors of healthy and infected plants in the current study.

In parasitoid performance assays we observed significantly higher rates of successful parasitoid development following wasp oviposition into aphid hosts feeding on CMV-infected rather than healthy plants, suggesting that indirect effects of CMV on aphid quality as hosts for the parasitoid is a major factor contributing the higher incidence of parasitism observed on infected plants (Fig. 3). Furthermore, the results of assays in which we switched aphids between infection treatments following wasp oviposition indicate that the aphid diet during the period of parasitoid larval development is a key factor.

These results contrast with the previous findings of Christiansen-Weniger *et al.*^46^ regarding the effects of the persistently transmitted *Barley yellow dwarf virus* (BYDV) on parasitoid success. Those authors found that parasitoid (*Aphidius ervi*) larval development was slowed in aphid hosts reared on virus-infected plants prior to parasitization, and that parasitized aphid hosts carrying the virus had higher mortality than those without the virus. The acquisition of persistently transmitted viruses such as BYDV by aphids (and other vector insects) requires more prolonged feeding on infected plants than does the acquisition of non-persistently transmitted viruses like CMV (hours to days vs. seconds to minutes). Once acquired, however, persistently transmitted viruses typically travel from the insect gut into the hemocoel, and thence to other host tissues (e.g., the salivary glands in aphids) from which they can be inoculated into new hosts. Consequently, the more intimate interactions of persistently transmitted viruses with their insect vectors may offer increased latitude for direct effects on insect physiology that are relevant for the success of parasitoids. Thus, while BYDV typically increases host plant quality for aphids, Christiansen-Weniger *et al.*^46^ speculated that direct virus effects on aphid physiology lead to increased mortality of parasitized aphids. However, another persistently transmitted virus, *Pea enation mosaic virus*, was reported to have no effect on parasitism rates when circulating within its aphid vector, *Acyrthosiphon pisum*^36^, suggesting that the *direct* effects of persistently transmitted viruses on the establishment and development of a larval parasitoid within an aphid host may vary depending on the virus-host interaction. The current findings demonstrate that viruses can also have positive effects on parasitoid success rate, and that such effects can be mediated by virus effects on host-plants, and thus on the diet of the aphid host, even for non-persistent viruses such as CMV that do not directly influence aphid physiology.

One of the most apparent ways in which a non-circulating plant virus, such as CMV, might indirectly influence parasitoid success is through changes in plant nutritional quality. Numerous studies demonstrate that variation in the quality and availability of food influences immune parameters in insects (e.g., 42,43,44,47,48,49,50) and that immune defenses in invertebrates are costly to maintain (reviewed in^51^). In earlier work on the present system we reported that CMV disrupts ratios of carbohydrates (glucose, fructose, and sucrose) to free amino acids in the phloem (the main feeding site for aphids) of *C. pepo* and also reduces the relative percentage of several essential and non-essential amino acids in the phloem, thus reducing the nutritional quality of the host for aphids^24^,^30^. Aphids do not undertake protein digestion^52^, so free amino acids and simple carbohydrates constitute the major nutrients in their diet. To compensate for overall low levels of essential amino acids in plant phloem, aphids contain obligate symbiotic bacteria (*Buchnera aphidicola*), which synthesize essential amino acids from more abundant precursors that are present in the diet or synthesized by the aphid itself from other ingested nutrients (e.g. carbohydrates)^53^,^54^. If the concentration of key free amino acids and simple carbohydrates in host-plant tissues is reduced by CMV infection, aphids may come under nutritional stress as their intake of both essential amino acids *and* their precursors are reduced.

In the current study we observed a significant difference in the early establishment of parasitoids (e.g. successful parasitism vs. unsuccessful parasitism) but did not see treatment differences in initial survival of aphid hosts after parasitoid attack. Nor did we observe differences in the emergence rate of parasitoids from successfully parasitized individuals (Fig. 3). The most likely explanation for the increased success of parasitoids on hosts feeding on CMV-infected plants is therefore that the ability of aphids to resist the initial stages of parasitoid egg hatching and development were compromised by dietary insufficiencies. This hypothesis is further supported by the fact that nutrient deprivation immediately following parasitism (i.e., when aphids are moved from healthy to infected plants at the time of was oviposition) is sufficient to elicit increased susceptibility to parasitoid oviposition. This finding is similar to the previous observation that *Drosophila melanogaster* individuals exhibit attenuated immune defenses (encapsulation ability) when deprived of yeast nutrients as a component of the diet immediately following oviposition by a cynipid wasp parasitoid^42^. The specific immune responses of *M. persicae* and other aphid species to parasitoid eggs or larvae (and indeed, to most antagonists) are not well characterized, but likely involve cellular immunity-based defenses (encapsulation)^55^. Recent work also suggests that aphid resistance to parasitoids may be mediated to a large extent by the presence of facultative bacterial endosymbionts (reviewed in^56^). In the present study we did not quantify changes in innate or endosymbiont-conferred immune parameters that may be compromised due to virus-induced changes in host quality. However, given that immune defenses and endosymbionts can both be costly to aphids^57^, it seems logical that host plant quality will be an important determinant of overall aphid immunity. Future work could usefully build on our current results by measuring immune responses or manipulating facultative endosymbionts in the context of variation in host-plant quality.

Regardless of the mechanisms by which CMV indirectly affects parasitoid success, our current findings likely have considerable relevance for understanding the spread of non-persistently transmitted viruses by vectors. Recent research by Hodge *et al.*^37^ and Dáder *et al.*^58^ provides strong evidence that parasitoids can influence (and increase) the transmission rates of non-persistently transmitted viruses by promoting aphid movement and dispersal, even as aphid longevity and overall population size is reduced by parasitism. Movement, probing, and migration between plants after dropping are all important components of virus spread, especially for non-persistent viruses that must be re-acquired frequently in order to be effectively transmitted^6^,^59^. As noted above, our current results suggest that foraging parasitoids exhibit similar attraction to odor cues from CMV-infected and uninfected plants (Fig. 2), and we have previously observed relatively similar overall rates of predator recruitment to healthy and infected plants in field studies^30^. These observations, together with the higher rates of parasitoid emergence from aphids feeding on infected plants observed in the current study (Figs. 1 and 3) suggest that disturbance by parasitoids and predators may be a significant factor prompting the dispersal of viruliferous aphids from infected plants. As a result, top-down effects of parasitoids on aphid populations might also be expected to exert significant selection pressure on the virus, selecting for variants that are able to alter host-plant physiology to influence tri-trophic plant-aphid-parasitoid interactions in ways conducive to the spread of the pathogen. In the case of CMV, the same virus-induced changes in host plant phenotype that favor attraction, probing, and dispersal of aphid vectors^15^,^17^,^24^ may also enhance the attraction of parasitoids to infected plants (i.e., via the elicitation of elevated volatile emissions that compensate for the diminished size of infected plants) and support parasitoid development on aphid hosts that do not choose to leave infected plants (e.g., nymphal instars, which are less prone to dispersal in the absence of disturbance). Such effects may not only ensure that aphid vectors experience periodic disturbance on virus-infected plants, but may also check aphid population growth on these plants, reducing plant mortality and increasing the amount of time that an infected plant will persist in the landscape as a source of inoculum. Future experiments in this and similar systems should explore the consequences of virus-induced changes in host plant quality and chemical phenotype for virus spread by studying transmission in semi-natural settings that incorporate parasitoids in the context of spatial and temporal factors that are altered by virus-induced changes in plant phenotype (long-range foraging cues and vector population dynamics).

## Methods

### Plant/insect culture and virus inoculations

Cultivated squash (*Cucurbita pepo* cv. ‘Dixie’, Willhite Seeds Inc.) were grown in square pots (1000 cm^3^) in sterile ProMix potting soil containing 5 g of slow-release fertilizer (Osmocote 14-14-14 N-P-K) and trace micronutrients (Scott’s Micromax Micronutrients) in an insect-free walk-in growth chamber (16:8 light:dark photoperiod; 23 °C day / 21 °C night). Plants at the cotyledon stage were inoculated with the equivalent of 5 cm^2^ of frozen stock tissue infected with CMV-Fny (stored at –80 °C). Frozen tissue was ground then combined with 15 mL of 0.1 M potassium phosphate buffer and fine-grit carborundum powder, then applied to cotyledon surfaces using cotton swabs. All inoculations occurred from the same stock collection of frozen tissue. Plants designated for the “healthy” treatment were given “mock inoculations”, using a solution containing tissue from a healthy squash plant. Plants were used for experiments when they were 3–4 weeks old.

*Myzus persicae* were raised in colonies on 4-week-old *Capsicum annuum* (cv. California Wonder) under ambient seasonal photoperiod. Colonies were refreshed weekly by moving 2-3 aphid-infested leaves (approximately 500-1,000 aphids) to a new plant in a clean cage. Parasitoids (*Aphidius colemani*) were purchased as mummies from Arbico Organics and reared on *M. persicae* maintained on *Capsicum annuum* but kept separate from the main *M. persicae* colony. Wasps fed on honeydew from the aphid hosts supplemented with a dilute mixture of honey, yeast, vitamin C, and water delivered on paper-towel wicks.

### Parasitoid foraging under semi-natural conditions

Small colonies of *M. persicae* were established on the most recently expanded two leaves of infected and healthy plants (10 treatment pairs) and allowed to grow for 10 days—in order to produce offspring that had developed entirely on the infected or healthy treatment. On the day of the foraging assay, 20-35 nymphs (3^rd^ instar) were removed from each plant of one pair onto the most recently expanded leaf of a new plant of the same treatment (creating a newly infested pair) using a fine paintbrush (each plant in a given pair always received the same number of nymphs with the total number varying somewhat due to the availability of 3^rd^ instar nymphs). Care was taken to move nymphs only after they had withdrawn their stylets and begun walking on the plant in order to avoid damaging their mouthparts. Nymphs were allowed to resume normal feeding and behavior on the new plants for several hours prior to experiments, but were confined to the leaf on which they had been released with a mesh sleeve.

Infested treatment and control plants (one plant per treatment) were placed at one end of a 1 m^3^ fine-mesh cage. The infested leaves were exposed from their sleeves in order to allow wasps access to the aphid hosts, but the plants were far enough apart (0.3 m) to prevent movement of nymphs between treatments (if nymphs did attempt to leave their leaf, they would contact the mesh sleeve and be re-directed back to the starting leaf). Sixteen female and 4 male wasps were then released from a vial at the opposite side of the cage (female wasps were likely to have mated in the colony prior to the experiments, but males were included to further ensure mating opportunities). Wasps were allowed to forage for 22 hours (overnight), beginning in the late afternoon. Plants were then removed and the mesh sleeve was pulled back up around each infested leaf. Nymphs remained on the plants for two weeks following the foraging assay (in a greenhouse with natural and supplemental lighting; 16:8 photoperiod at an average temperature of ~25°C), during which time mummy development and wasp emergence were tracked. We calculated the proportion of nymphs parasitized (i.e., becoming mummies) out of the total number exposed within each infection treatment across all tests (N = 193) and compared these proportions using a two-tailed Z-test. Emergence was calculated as a proportion of the total number of mummies for each infection treatment (N = 92 infected; N = 44 healthy) and these proportions were also compared using a two-tailed Z-test (significance level: P < 0.05).

### Odor-based foraging assays

Small colonies of *M. persicae* (approximately 10 adults) were started on 6 treatment pairs of plants and reared in a growth chamber (25 °C; 16:8 photoperiod). Shortly after founding, colonies were standardized to include only 30 3^rd^ instar nymphs (i.e., adults and other instars were removed)—in order to mimic the infestations employed for plants in the large-cage foraging assays. The upper portion of each plant (5-6 most recently expanded leaves, including the infested leaf) was enclosed in a 4 L glass dome fitted with ports that allowed the input of charcoal-filtered, humidified air (1 L per minute) and the movement of air from each dome into one of the two arms of the Y-tube olfactometer (base: 27 cm, arms: 18 cm, diameter: 2.5 cm). Domes were artificially illuminated to allow photosynthesis and volatile production, but the Y-tube was positioned vertically in an opaque box to remove the influence of light on wasp choice. The lower base of the Y-tube was also fitted with a tube that pulled a slight vacuum (0.25 L per minute), facilitating the flow of air from the domes, through the arms, and down the base to the release point for the wasp. Prior to use of this assay with treatment pairs, the set-up was tested to ensure that wasps could discriminate among targets known to be attractive (aphid-infested vs. uninfested plants or an empty chamber).

Six pairs of plants were used, and for each pair we tested 12-16 female wasps that were 2–4 days old (wasps were removed from the colony 24 hours prior to tests and supplied with water and food in the interim). For each trial, a single wasp was released at the base of the Y-tube in a small chamber and was given 5 minutes to make a choice, which entailed either entering the chamber at the terminal end of the olfactometer arm, or passing the half-way point of the arm and remaining beyond it for more than 1 minute (wasps that failed to make a choice typically did not leave the starting chamber). After every 4 trials, the Y-tube olfactometer was rinsed with acetone and hexanes and the treatments were switched between arms of the olfactometer. Trials were run for 92 total wasps, of which 13 failed to make choices and were excluded from the analysis. Data were analyzed using a Chi-squared test (significance level: P < 0.05).

### Parasitism success rates following oviposition

Colonies were established on infected and healthy plants (3 pairs) as above and allowed to grow for 10 days. From each plant 20-30 3^rd^ instar aphids were removed, one at a time, by carefully excising the plant tissue on which they were feeding. Each aphid was placed in a small, glass-enclosed arena with a pool of 5-8 female wasps. Aphids were observed until each received at least one “deep sting” (as opposed to superficial probing) from a wasp’s ovipositor. This stinging behavior is indicative of an oviposition event that also includes the injection of wasp-derived components capable of influencing aphid immunity and physiology^60^. Parasitized aphids were then placed on new infected or healthy plants with leaves enclosed in cages. To enable tracking of aphids over time while mimicking the normally gregarious colonization habit of aphids, groups of approximately 10-16 parasitized aphids were established on each leaf, 6 groups for hosts on infected plants, and 5 for hosts on healthy plants. Aphid survival was assessed after 3 days, and parasitoid mummy development was assessed from day 10 to day 14 post oviposition. In the intervening period, aphids were observed daily and any newly produced first instar nymphs were removed. For each infection treatment we measured the proportion of aphids surviving attack out of the total exposed to oviposition (N = 72 infected; N = 62 healthy) and the proportion of survivors that developed into mummies (N = 60 infected, N = 45 healthy survivors). Proportions for both categories were compared for the two infection treatments using two-tailed Z-tests (significance level: P < 0.05).

We performed a second, similar experiment in which, after parasitism, aphids reared on infected plants were placed onto healthy plants (5 populations [12-26 aphids per population] across 5 plants), while aphids reared on healthy plants were placed onto infected plants (5 populations [12-23 aphids per population] across 5 plants). Survival was assessed after 2–3 days, and parasitoid mummy development was assessed from day 10 to day 14 post oviposition. The proportion of aphids from each treatment regime surviving attack, out of the total exposed to oviposition (N = 85 healthy to infected; N = 89 infected to healthy), and the proportion of survivors developing into mummies (N = 62 healthy to infected; N = 65 infected to healthy) were compared using two-tailed Z-tests (significance level: P < 0.05).

## Additional Information

**How to cite this article**: Mauck, K. E. *et al.* Infection of host plants by Cucumber mosaic virus increases the susceptibility of *Myzus persicae* aphids to the parasitoid *Aphidius colemani*. *Sci. Rep.* **5**, 10963; doi: 10.1038/srep10963 (2015).
